# Efficacy of Platelet-Rich Plasma Dressing Versus Normal Saline Dressing in the Management of Diabetic Foot Ulcers: A Comparative Study Conducted in a Tertiary Care Hospital in Southern Odisha

**DOI:** 10.7759/cureus.87106

**Published:** 2025-07-01

**Authors:** Lavanya Jain, Manoj K Sethy, Anil K Jena, Abinash Pattanaik, Rajinder K Mishra, Madhumita Bhakta, Abhimanyu Behera

**Affiliations:** 1 General Surgery, Maharaja Krishna Chandra Gajapati (MKCG) Medical College and Hospital, Berhampur, IND; 2 Community Medicine, Maharaja Krishna Chandra Gajapati (MKCG) Medical College and Hospital, Berhampur, IND

**Keywords:** diabetic complications, diabetic foot ulcers management, normal saline dressing, platelet-rich plasma/ prp, ­wound healing

## Abstract

Background and objectives

Diabetic foot ulcers (DFUs) present significant challenges due to their chronicity and potential complications. This study aims to compare the efficacy of platelet-rich plasma (PRP) versus normal saline dressing (NSD) in promoting wound healing and reducing complications among diabetic patients.

Materials and methods

This comparative study enrolled diabetic patients with foot ulcers, randomly assigned to either PRP or NSD treatment groups. Demographic data, ulcer characteristics, and treatment outcomes were meticulously recorded and analyzed. Statistical tests, including paired and independent samples t-tests, chi-square tests, and descriptive statistics, were employed to assess differences between the groups.

Results

Both groups exhibited similar age distributions, with PRP showing a slightly wider range. Sex distribution was identical between the NSD and PRP groups. PRP therapy demonstrated significant wound size reduction compared to NSD, with notable improvements in ulcer healing rates and reduced rates of complications and surgical interventions. Microbiological analysis revealed comparable infection rates between groups, with PRP potentially reducing infection-related complications.

Interpretation and conclusion

PRP therapy appears to offer superior efficacy over NSD in promoting DFU healing, as evidenced by substantial reductions in ulcer size and lower rates of complications. This study supports PRP as a promising treatment modality for DFUs, potentially enhancing clinical outcomes and reducing healthcare burdens.

## Introduction

Diabetic foot ulcers (DFUs) are among the most serious complications of diabetes mellitus, contributing significantly to patient morbidity, lower extremity amputations, and escalating healthcare costs globally. The prevalence of DFUs is estimated at approximately 15% among individuals with diabetes, with a substantial risk of infection, delayed healing, and limb loss if not managed appropriately [1]. Chronic hyperglycemia in diabetes induces neuropathy, ischemia, and impaired immune responses, all of which synergistically hinder wound healing and increase vulnerability to infections [2].

Effective wound care strategies are therefore essential to reduce morbidity and enhance clinical outcomes in diabetic foot management. Normal saline dressing (NSD) remains a widely used conventional method due to its non-irritating and cost-effective properties. However, it lacks bioactive components that are critical for stimulating and accelerating tissue repair [3].

Recent advances in regenerative medicine have highlighted the therapeutic potential of autologous platelet-rich plasma (PRP) in enhancing wound healing. PRP, derived from the patient’s own blood, is a concentrated source of platelets rich in growth factors such as platelet-derived growth factor (PDGF), transforming growth factor-beta (TGF-β), and vascular endothelial growth factor (VEGF). These factors collectively promote angiogenesis, fibroblast proliferation, and extracellular matrix remodeling [4]. As such, PRP represents a promising alternative to conventional treatments for DFUs, with the potential to reduce healing time and lower the likelihood of surgical intervention [5].

Despite its promise, PRP therapy remains underutilized in diabetic foot care, primarily due to inconsistencies in preparation methods, concerns over cost, and the absence of standardized treatment protocols. Existing studies have reported mixed findings: while some demonstrate that PRP accelerates wound closure and reduces infection rates, others have found negligible benefits compared to standard wound care [6]. These inconsistencies underscore the need for further comparative research to validate its efficacy and define its clinical role.

The present study aims to compare the effectiveness of PRP therapy versus NSD in the management of DFUs, focusing on wound healing parameters, ulcer size reduction, infection control, and the incidence of complications or need for surgical procedures. The primary objective is to determine whether PRP results in a significantly greater percentage of ulcer size reduction and improved healing outcomes compared to NSD in early-grade ulcers. Secondary objectives include assessing differences in bacterial colonization, glycemic control, and the rate of surgical complications between the two groups. By generating empirical evidence on the relative efficacy of PRP, this study seeks to inform best practices for DFU management and improve overall patient care in diabetic foot settings.

## Materials and methods

This single-center, prospective, hospital-based comparative study was conducted in the Department of General Surgery at Maharaja Krishna Chandra Gajapati (MKCG) Medical College and Hospital, Berhampur, Odisha, over a duration of 21 months, from June 2023 to February 2025. The primary objective was to evaluate and compare the efficacy of PRP dressing with conventional NSD in the management of chronic DFUs among patients attending the outpatient and inpatient departments of this tertiary care center. The sample size was calculated using the formula for comparing two means:

n = [( Z _1-α/2_ +Z_1-β_ )/(Δ/σ)]^2^ × 2

where Z_1-α/2 _= 1.96 for a 95% confidence level, Z_1-β _= 0.84 for 80% power, and the expected effect size (Δ/σ) was 0.79. 

Based on preliminary inputs and statistical considerations, a total sample size of 50 participants - 25 in each group - was determined to be adequate to achieve sufficient statistical power for detecting meaningful differences in outcomes. Participants were selected through simple random sampling, and allocation to treatment groups was conducted using a computer-generated randomization sequence. This ensured unbiased assignment into either the PRP group (Group A) or the NSD group (Group B). Eligibility criteria included patients aged between 12 and 75 years, diagnosed with either Type I or Type II diabetes mellitus, and presenting with chronic foot ulcers that had persisted for more than four weeks. The ulcer size had to be less than 10 × 10 cm, and fasting blood glucose levels were required to fall between 140 mg/dL and 200 mg/dL, measured on two separate occasions within a 24-hour period. Exclusion criteria encompassed the presence of pulseless limbs, immunocompromised status, osteomyelitis, cellulitis, malignancies involving the ulcer area, diabetic ketoacidosis, or ulcers exposing bone or tendons. These stringent criteria were applied to ensure a homogenous, clinically stable population suitable for assessing the local therapeutic effects of the interventions.

Following written informed consent, the 50 eligible participants were randomized into the two intervention arms. Group A received PRP dressings, while Group B received conventional NSD, each administered biweekly for four weeks. PRP was prepared using a standardized double-spin centrifugation technique from 20 mL of autologous whole blood, following established procedural protocols to ensure consistency in platelet concentration and growth factor content. The primary outcome measure was the percentage reduction in wound size, evaluated using a transparent measurement grid at baseline and during weekly follow-up visits. Secondary outcomes included the time to complete wound healing, incidence of infection, and the occurrence of any local complications such as necrosis, abscess formation, or worsening ulcer characteristics. Data were systematically recorded using structured case record forms (CRFs) and entered into Microsoft Excel (Microsoft® Corp., Redmond, WA) for analysis. Statistical analyses were performed using jamovi version 2.3.28 (Jonathon Love, Damian Dropmann, and Ravi Selker, Sydney, Australia). Quantitative variables were analyzed using independent t-tests, while categorical variables were assessed using chi-square tests. A p-value < 0.05 was considered statistically significant. To maintain data accuracy and procedural standardization, all study personnel underwent prior training sessions on data collection, wound measurement techniques, and dressing protocols. Periodic audits were conducted, and data entry accuracy was cross-verified by senior faculty members. The study received ethical clearance from the Institutional Ethics Committee of MKCG Medical College and Hospital, under approval number 1370. All participants were assured of confidentiality, voluntary participation, and adherence to the principles of non-maleficence and beneficence, in accordance with the Indian Council of Medical Research (ICMR) Ethical Guidelines and the Declaration of Helsinki.

## Results

The sociodemographic characteristics of the study participants (Table 1) were largely comparable between the PRP and NSD groups, with some minor variations.

**Table 1:** Distribution of study population based on sociodemographic profile of study participants (N = 50) Table showing the distribution of the study population (N = 50) according to sociodemographic characteristics, including variables such as age, gender, education, occupation, socioeconomic status, and residence. This categorization helps in understanding the background profile of the participants involved in the study. NSD - normal saline dressing, PRP - platelet-rich plasma

Sociodemographic profile		Group	
		NSD	PRP
Age (mean ± SD)		53.1 ± 8.36	52.2 ± 9.31
Sex	Female	10 (20%)	10 (20%)
	Male	15 (30%)	15 (30%)
Address	Rural	12 (24%)	16 (32%)
	Urban	13 (26%)	9 (18%)
Education	Illiterate	3 (6%)	5 (10%)
	Literate	22 (44%)	20 (40%)
Socioeconomic status	Lower	6 (12%)	11 (22%)
	Lower-middle	8 (16%)	9 (18%)
	Upper-middle	11 (22%)	5 (10%)

The mean age of participants in both the PRP and NSD groups was comparable, although the NSD group exhibited a slightly higher average age and lower variability in age distribution, suggesting a more uniform age profile among its participants. Of the total sample size (N = 50), 30 participants (60%) were male and 20 participants (40%) were female, reflecting a reasonably balanced sex distribution across both study arms.

In terms of residential background, a higher proportion of participants in the PRP group (16 individuals; 32%) were from rural areas, compared to 12 individuals (24%) in the NSD group. Conversely, the NSD group had a larger share of urban participants (13 individuals; 26%) compared to the PRP group (nine individuals; 18%). This pattern suggests a slight predominance of rural representation overall in the study population. Literacy levels were broadly comparable between the two groups. The PRP group included a slightly higher number of illiterate participants, whereas the NSD group had a marginally higher number of literate individuals, indicating a modest difference in educational background between the cohorts. The sample also captured a diverse range of occupational categories. The most represented groups included homemakers (11 participants; 22%), business professionals (10 participants; 20%), and service workers (10 participants; 20%). Other occupations, such as contractors, domestic workers (maids), and teachers, were minimally represented, reflecting a varied but uneven occupational distribution.

With respect to socioeconomic status, the PRP group had a greater proportion of participants from lower socioeconomic backgrounds (11 individuals; 22%) compared to 6 individuals (12%) in the NSD group. In contrast, the NSD group included a larger number of participants from the upper middle class (11 individuals; 22%) as opposed to five individuals (10%) in the PRP group. These differences indicate a moderate socioeconomic disparity between the two groups, which may influence access to or perceptions of healthcare interventions.

Diabetic neuropathy was present in a substantial proportion of the study population, with 36 out of 50 participants (72%) affected (Table 2). Among the two groups, the NSD group had a slightly higher prevalence of neuropathy (19 participants; 38%) compared to the PRP group (17 participants; 34%). Conversely, a greater number of participants in the PRP group were free from neuropathy (eight participants; 16%) compared to the NSD group (six participants; 12%), suggesting a marginal difference in baseline neurological status between the groups. Assessment of ulcer etiology revealed that traumatic ulcers were more common than spontaneous ulcers in both groups. In the NSD group, 17 participants (34%) presented with traumatic ulcers, while 15 participants (30%) in the PRP group had similar findings. However, the PRP group had a slightly higher occurrence of spontaneous ulcers (10 participants; 20%) compared to eight participants (16%) in the NSD group, indicating a marginally different ulcer origin profile. Regarding ulcer location, plantar ulcers were predominant in both groups, with 16 participants (32%) in the NSD group and 15 participants (30%) in the PRP group presenting with ulcers on the sole of the foot. Meanwhile, dorsal ulcers were slightly more frequent in the PRP group (10 participants; 20%) compared to the NSD group (nine participants; 18%), suggesting a similar anatomical distribution overall, with a slight variation in dorsal involvement.

**Table 2:** Surgical history and wound parameters (N = 50) Comparison of clinical and treatment-related parameters between NSD and PRP groups among diabetic foot ulcer patients (N = 50). The table includes data on diabetic neuropathy status, mode of ulcer onset, site of ulcer, type of anti-diabetic treatment, and changes in ulcer area (mean ± SD) from initial to final measurements. NSD - normal saline dressing, OHA - oral hypoglycemic agent; PRP - platelet-rich plasma

Factors		Group	
		NSD	PRP
Diabetic neuropathy	Absent	6 (12%)	8 (16%)
	Present	19 (38%)	17 (34%)
Ulcer onset	Spontaneous	8 (16%)	10 (20%)
	Traumatic	17 (34%)	15 (30%)
Site	Dorsal	9 (18%)	10 (20%)
	Plantar	16 (32%)	15 (30%)
Anti-diabetic treatment	Injectable	20 (40%)	19 (38%)
	OHA	5 (10%)	6 (12%)
Initial area in mm^2^ (mean ± SD)		48 ± 9.43	51 ± 11
Final area in mm^2^ (mean ± SD)		41 ± 8.22	33.4 ± 6.92
Area change in mm^2^ (mean ± SD)		6.98 ± 2.54	17.6 ± 4.72

**Figure 1:**
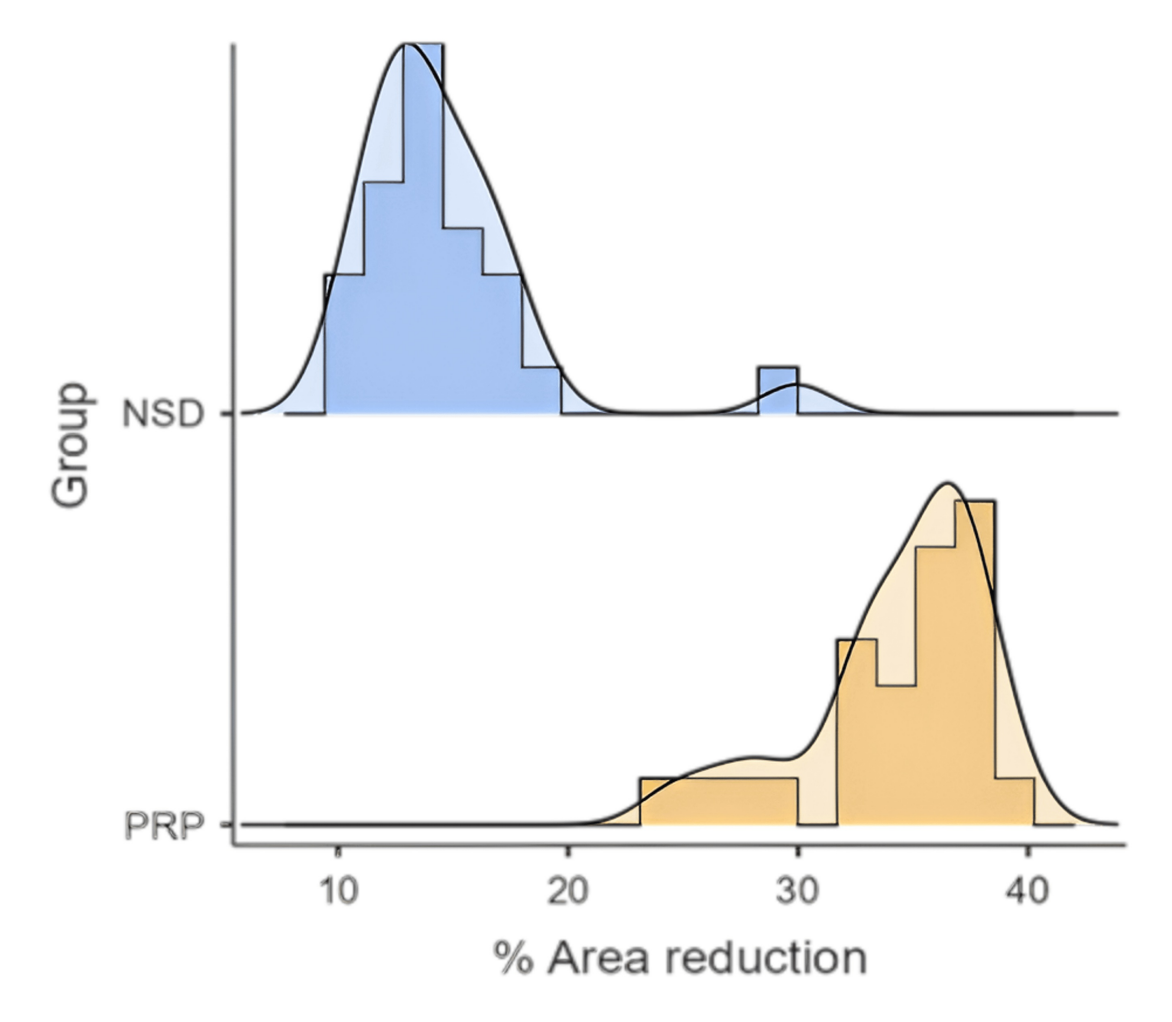
Distribution of study population based on percentage of area reduction (N = 50) Figure showing the distribution of the study population (N = 50) based on the percentage of ulcer area reduction over the study period. This parameter reflects the effectiveness of the treatment interventions in promoting wound healing among participants. NSD - normal saline dressing, PRP - platelet-rich plasma

In terms of diabetes management, the majority of participants in both groups were receiving injectable insulin therapy - 20 participants (40%) in the NSD group and 19 participants (38%) in the PRP group. The use of oral hypoglycemic agents (OHAs) was less common, reported in five participants (10%) from the NSD group and six participants (12%) from the PRP group. This indicates that the majority of the study population was on more intensive glycemic control regimens. When analyzing previous surgical history, a higher proportion of PRP participants (11 individuals; 22%) reported undergoing prior surgical interventions related to their condition compared to the NSD group (eight individuals; 16%). On the other hand, a greater number of NSD participants (17 individuals; 34%) had no prior surgical history, compared to 14 individuals (28%) in the PRP group. This may suggest a higher baseline severity or chronicity in the PRP group. In terms of clinical outcomes, PRP therapy demonstrated significantly improved wound healing compared to conventional NSD. The mean reduction in ulcer area was markedly greater in the PRP group, with a mean decrease of 17.6 mm^2^, whereas the NSD group showed a much smaller reduction of only 6.98 mm^2^. This substantial difference reinforces the superior efficacy of PRP in promoting tissue regeneration and wound closure. Notably, despite participants in the PRP group presenting with slightly larger initial ulcer sizes, they still achieved a greater absolute reduction in wound area, further supporting the enhanced healing potential of PRP (Figure 1).

Statistical analysis strongly supported the superior wound-healing efficacy of PRP therapy over NSD. The paired samples t-test demonstrated a statistically significant reduction in ulcer area across the entire sample (t = 13.3, degrees of freedom = 49, p < 0.001), confirming that both treatment modalities were effective in reducing wound size over time. However, comparative analysis using the independent samples t-test revealed that the PRP group achieved markedly better outcomes. Participants receiving PRP therapy exhibited significantly smaller final ulcer areas, a greater absolute reduction in ulcer size, and a higher percentage reduction in wound area compared to those in the NSD group (p < 0.001 for all parameters). These results clearly indicate that while both interventions contribute to healing, PRP is substantially more effective in accelerating wound closure and minimizing residual ulcer dimensions. Furthermore, the chi-square test identified a strong association between treatment modality and the incidence of surgical complications (χ² = 35.5, p < 0.001). Participants in the NSD group experienced a significantly higher frequency of complications, including infections requiring additional surgical intervention. In contrast, the PRP group had a markedly lower complication rate, suggesting not only better healing but also a reduced need for invasive follow-up procedures (Table 3). These findings underscore the clinical advantages of PRP therapy, both in enhancing wound healing and in lowering the risk of adverse outcomes, thereby supporting its integration into standard DFU management protocols.

**Table 3:** Comparison of wound healing outcomes and complications between NSD and PRP groups (N = 50) The table presents a comparative analysis of wound healing parameters and complication rates between patients treated with NSD and PRP. Variables include final ulcer area, area change, percentage area reduction, and complication occurrence. Statistical significance was evaluated using independent and paired t-tests for continuous variables and the chi-square test for categorical variables. A p-value of <0.001 indicates highly significant differences favoring the PRP group in terms of wound healing and reduced complications. NSD - normal saline dressing, PRP - platelet-rich plasma

Factors		Group		Statistical test	p-value
		NSD	PRP		
Final area in mm^2^ (mean ± SD)		41 ± 8.22	33.4 ± 6.92	Independent t-test	<0.001
Area change in mm^2^ (mean ± SD)		6.98 ± 2.54	17.6 ± 4.72	Independent t-test	<0.001
Initial area vs. final area (mean ± SD)		48 ± 9.43 vs. 41 ± 8.22	51 ± 11 vs. 33.4 ± 6.92	Paired t-test	<0.001
Percentage area reduction		14.5 ± 3.91	34.3 ± 3.82	Independent t-test	<0.001
Complications	No	1 (2%)	22 (44%)	Chi-square test	<0.001
	Yes	24 (48%)	3 (6%)		

**Figure 2:**
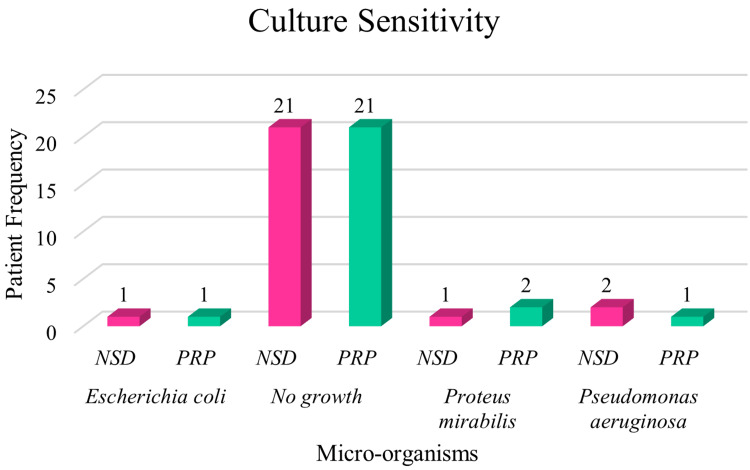
Distribution of study population based on microorganisms found in culture (N = 50) Figure showing the distribution of the study population (N = 50) based on microorganisms isolated from wound cultures. The data represent the frequency of different bacterial species identified, highlighting the microbiological profile associated with diabetic foot ulcers in the study group. NSD - normal saline dressing, PRP - platelet-rich plasma

Bacterial culture analysis revealed comparable findings between the PRP and NSD groups, indicating a similar microbiological profile across treatment modalities. Out of the total 50 wound samples analyzed, 21 samples (42%) showed no bacterial growth, suggesting a relatively low overall burden of wound infection within the study population (N = 50). Among the positive cultures, only minimal differences were noted in the distribution of specific bacterial pathogens. Escherichia coli was isolated in one case (2%) in each group, reflecting identical prevalence. Proteus mirabilis was detected in one participant (2%) in the NSD group and two participants (4%) in the PRP group (N = 25 per group), indicating a slight but clinically negligible variation. Similarly, *Pseudomonas aeruginosa* was found in two cases (4%) in the NSD group and one case (2%) in the PRP group, again showing no meaningful difference in pathogen distribution (N = 25 per group). These results demonstrate that the rate and type of bacterial colonization were broadly comparable between both treatment arms, with no statistically significant disparities in infection rates. This suggests that PRP therapy’s superior wound healing outcomes were not attributable to differences in baseline or acquired microbial infections, but rather to the biological properties of the intervention itself (Figure 2).

However, the NSD group experienced a significantly higher incidence of post-treatment complications and need for further surgical interventions, with 24 participants (48%) out of 25 requiring additional surgical procedures (N = 25 per group). In stark contrast, only three participants (6%) in the PRP group required similar surgical management, highlighting a statistically and clinically meaningful reduction in complication rates associated with PRP therapy (Table 3). Moreover, 22 participants (44%) in the PRP group successfully avoided the need for any further surgical procedures, underscoring the protective and therapeutic advantage of PRP in preventing the escalation of wound severity. Comparatively, only one participant (2%) in the NSD group managed to avoid additional interventions, reinforcing the limited effectiveness of standard saline dressings in arresting disease progression and facilitating complete wound resolution. These findings strongly suggest that PRP not only enhances the wound healing process but also significantly reduces the likelihood of complications and the necessity for surgical escalation, making it a more effective and safer alternative for managing chronic DFUs.

## Discussion

The findings of this study are consistent with existing literature highlighting the efficacy of PRP therapy in the management of DFUs. Prior studies have established that PRP accelerates wound healing by promoting angiogenesis, fibroblast proliferation, and extracellular matrix deposition, thereby enhancing tissue regeneration in chronic, non-healing wounds [7,8]. In this study, the significant reduction in ulcer size observed in the PRP group compared to the NSD group supports the findings of Asif et al. [9], who reported that wounds treated with PRP showed faster healing rates than those treated with conventional saline. Furthermore, a meta-analysis conducted by Martinez-Zapata et al. [10] substantiated that PRP substantially improves wound closure rates and reduces overall healing time in diabetic ulcer patients. From a demographic standpoint, the study observed a slight rural predominance, with 28 participants (56%) (N = 50) residing in rural areas. This aligns with reports by Rao et al. and Zhang et al. [11,12], who emphasized a higher prevalence of DFUs in rural populations, attributed to limited healthcare access, delayed presentation, and inadequate diabetes management. The sex distribution, consisting of 60% males and 40% females, mirrors the pattern identified by Margolis et al. [13], who associated higher DFU rates in males with increased physical activity, occupational exposure, and potentially lower healthcare-seeking behavior. The mean age of participants in both groups was similar and closely matched the age groups reported by Game et al. [14] and Armstrong et al. [2], who noted that DFUs are most prevalent in older adults due to longer disease duration, age-related neuropathy, and vascular compromise. Diabetic neuropathy was present in 36 participants (72%) (N = 50), a figure consistent with Boulton et al. [15], who estimated neuropathy prevalence in DFU patients between 60% and 80%. While the NSD group had a slightly higher prevalence (19 participants, 38%) compared to the PRP group (17 participants, 34%), the similarity indicates that both cohorts were comparable at baseline with respect to neuropathic risk factors. With regard to ulcer etiology, traumatic ulcers were more common than spontaneous ones in both groups (NSD: 17 (34%), PRP: 15 (30%)), reflecting trends identified by Lavery et al. [16], who observed trauma as a leading cause of ulceration in diabetics due to sensory deficits and impaired protective mechanisms. Additionally, plantar ulcers were more frequent than dorsal ulcers across both arms (NSD: 16 (32%), PRP: 15 (30%)), consistent with Singh et al. [17], who highlighted plantar pressure and repeated microtrauma as key risk factors for ulcer formation.

Importantly, the role of PRP in reducing the need for surgical intervention and minimizing complications was strongly evident. Only three participants (6%) in the PRP group required further surgical procedures, compared to 24 participants (48%) in the NSD group, demonstrating a substantial reduction in surgical burden. These results are in alignment with studies by Carter et al. [18] and Anitua et al. [19], which emphasized PRP’s ability to reduce amputation rates and limit the need for additional surgical care by enhancing tissue repair processes. The statistically significant association between treatment type and complication rate (χ² = 35.5, p < 0.001) further supports this, echoing the findings of Everts et al. [20], who documented PRP’s anti-inflammatory and infection-limiting effects. Although bacterial culture results did not reveal any significant differences between the groups - with 21 out of 50 samples (42%) showing no bacterial growth - this aligns with earlier studies by Frykberg et al. [21] and Saad Setta et al. [22], which postulated that PRP may possess intrinsic antimicrobial properties, potentially aiding in infection control even when overt bacterial colonization is absent. The statistical analysis reinforces PRP’s therapeutic superiority. The paired t-test (t = 13.3, p < 0.001) confirmed significant within-group reduction in ulcer area, while independent t-tests (p < 0.001) demonstrated that the PRP group achieved greater overall reductions in final ulcer size and percentage area healed. These findings are consistent with those of Lacci and Dardik [23], who showed that PRP facilitates more rapid and complete healing in chronic wounds. This study demonstrates the superior efficacy of PRP therapy over conventional NSD in the management of DFUs. PRP was associated with significantly greater ulcer size reduction (p < 0.001) and markedly lower complication rates, establishing it as a promising, biologically active intervention. The inclusion of a well-balanced sociodemographic cohort, systematic outcome assessment, and robust statistical analysis strengthens the internal validity of these findings.

However, several limitations merit attention. While the sample size (N = 50) was statistically adequate, a larger cohort would enhance the generalizability of results. The short follow-up duration limited the ability to assess long-term outcomes, including recurrence and durability of healing. Additionally, the cost-effectiveness and accessibility of PRP therapy were not evaluated, though they are critical for adoption in resource-limited settings. Variability in PRP composition due to individual donor characteristics and the absence of molecular assays to explore the precise biological mechanisms of healing represent areas for future investigation. These findings contribute valuable evidence supporting PRP as an effective therapeutic option in diabetic wound care and underscore the need for larger, multicentric, and longer-term studies to validate and standardize its clinical use.

## Conclusions

PRP therapy has been shown to significantly accelerate wound healing, reduce complications, and decrease the need for surgical intervention in patients with DFUs. These findings are consistent with existing literature, further supporting the potential of PRP as an effective therapeutic modality. Future research should aim to investigate long-term outcomes, assess cost-effectiveness, and explore the underlying biological mechanisms to optimize its clinical application and promote broader accessibility.
